# Effects of DementiaNet’s Community Care Network Approach on Admission Rates and Healthcare Costs: A Longitudinal Cohort Analysis

**DOI:** 10.34172/ijhpm.2023.7700

**Published:** 2023-10-28

**Authors:** Toine EP Remers, Florien M. Kruse, Simone A. van Dulmen, Dorien L. Oostra, Martijn FM Maessen, Patrick PT Jeurissen, Marcel GM Olde Rikkert

**Affiliations:** ^1^Radboud university medical center, Scientific center for quality of healthcare (IQ healthcare), Nijmegen, The Netherlands.; ^2^Radboud university medical center, Department of Geriatric Medicine, Nijmegen, The Netherlands.; ^3^Coöperatie Volksgezondheidszorg, Business intelligence services, Arnhem, The Netherlands.; ^4^Radboud university medical center, Donders Institute for Brain, Cognition and Behaviour, Department of Geriatric Medicine, Radboud Alzheimer Centre, Nijmegen, The Netherlands.

**Keywords:** Dementia, Community Care Networks, Healthcare Costs, Hospital Admissions, Health Policy, The Netherlands

## Abstract

**Background:** People with dementia are increasingly living at home, relying on primary care providers for most healthcare needs. Suboptimal collaboration and communication between providers could cause inefficiencies and worse patient outcomes. Innovative strategies are needed to address this growing disease burden and rising healthcare costs. The DementiaNet programme, a community care network approach targeted at patients with dementia in the Netherlands, has been shown to improve patient’s quality of care. However, very little is known about the impact of DementiaNet on admission risks and healthcare costs. This study addresses this knowledge gap.

**Methods:** A longitudinal cohort analysis was performed, using medical and long-term care claims data from 38 525 patients between 2015-2019. The primary outcomes were risk of hospital admission and annual total healthcare costs. Mixed-model regression analyses were used to identify changes in outcomes.

**Results:** Patients who received care from a DementiaNet community care network showed a general trend in lower risk of admission for all types of admissions studied (ie, hospital, emergency ward, intensive care, crisis, and nursing home). Also, the intervention group showed a significant reduction of 12% in nursing days (relative risk [RR] 0.88; 95% CI: 0.77– 0.96). No significant differences were found for total healthcare costs. However, we found effects in two sub-elements of total healthcare costs, being a decrease of 19.7% (95% CI: 7.7%–30.2%) in annual hospital costs and an increase of 10.2% (95% CI: 2.3%–18.6%) in annual primary care costs.

**Conclusion:** Our study indicates that DementiaNet’s community care network approach may reduce admission risks for patients with dementia over a long-term period of five years. This is accompanied by a decrease in nursing days and savings in hospital care that exceed increased primary care costs. This improvement in integrated dementia care supports wider scale implementation and evaluation of these networks.

## Background

**Key Messages**

**Implications for policy makers**

- This study finds that local community-based care networks created under the DementiaNet programme lower both admission risks and hospital care costs for people with dementia, and show such effects are maintained over a long-term period of up to five years.
- Combined with previously demonstrated improvements in quality of care, the DementiaNet program has great potential to improve dementia care.
- Further implementation of DementiaNet’s community care network approach, both nationally and internationally could therefore assist the transition of current fragmented dementia care into integrated care processes.
- As the composition and functioning of care networks strongly depends on health system characteristics, international settings need to tailor these community care networks to their different systems.

**Implications for the public**

To this day, the organisation of dementia care remains suboptimal in many countries, with fragmentation being one of the main challenges. Patients with dementia are often treated by a wide range of healthcare professionals and providers, making effective communication difficult. Improving communication and collaboration between the different care providers can improve the wellbeing and health of patients and can lessen the care burden for informal caregivers. The findings of this study show that community care networks for patients with dementia created within the DementiaNet programme can both be cost-effective – potentially lowering healthcare costs and improve health outcomes in terms of admission risks and nursing days.

Patients with dementia often show complex patterns of multimorbidity, involving various healthcare organisations and professionals, which makes coordination difficult and hinders cost-effective care.^1^ Additionally, an increasing share of patients remains living at home, leading to additional challenges in care coordination and a potential increased risk of urgent admissions for people with advanced dementia in case such home-based care is not properly provided and coordinated.^2^ Ultimately, this results in higher mortality, reduced quality of life, and increased healthcare expenditures for this patient group.^3,4^ Better integration of care is key to bending this cost curve and decreasing admission risks whilst increasing quality of care.^2,5,6^

 Case management strategies with one dedicated care professional have been shown to be beneficial in this regard,^7^ but such strategies are likely to leave other healthcare providers unaware of each other’s care activities and of the health status of their shared patients. Also, case management strategies can be relatively expensive and are probably not sustainable given the growing staff shortages in healthcare.^8,9^ Alternatively, general practitioners (GPs) have been shown to deliver post-diagnosis dementia care that is of equal quality and cost-effectiveness compared to specialized memory clinics.^10,11^ Primary care professionals could therefore potentially play a more prominent role in facilitating high-quality integrated care for patients with dementia; a strategy that has already been shown to be cost-effective for occupational therapy.^12^

 The DementiaNet programme is an integrated network-based approach that was implemented in 2015 in the Netherlands to enable this transition towards primary care-based integrated dementia care.^13^ With the DementiaNet programme, local community care networks were formed to facilitate interprofessional collaboration between primary healthcare professionals from medical, care, and social domains who are caring for the same patients.^13^ Four key elements apply to each network: (1) a transition towards network-based care; (2) appointment of one or two dedicated network leaders; (3) network goal-setting for quality improvement purposes; and (4) interprofessional training on relevant self-chosen topics.^13^ In total, 40 networks have been formed so far. Results showed improvements in networks’ quality of care and interprofessional collaboration over a period up to six years.^14-16^

 A comparable community network programme targeted at patients with Parkinson’s disease was among the first to show that a such an approach can be beneficial for health outcomes and costs over a three-year period.^17^ However, thus far, it has not yet been studied if these improvements in quality of care within the DementiaNet programme have led to reduced admission risks and healthcare costs. Also, evidence on these outcomes for other dementia-specific programmes remains inconclusive as a result of varying study periods and small study samples.^18-20^ Moreover, the available studies look at hospital-initiated network programmes instead of primary care-based community network approaches.^18-20^ Therefore, the aim of this study is to determine the long-term impact on admission risks and healthcare costs of DementiaNet’s community care network approach.

## Methods

###  Study Design and Data

 We performed a retrospective longitudinal cohort analysis on routinely-collected claims data from a large cohort of Dutch inhabitants insured by cooperation ‘Volksgezondheidszorg’ (VGZ), one of the largest non-profit insurance companies in the Netherlands with a market share of 24.3%.^21^ The majority of healthcare in the Netherlands is delivered through two coexisting schemes: curative care and long-term care (see Box 1). Compulsory and automatic enrolment within both schemes ensures that our data contains most health claims of insured patients with dementia. Our data includes claims for curative care from January 1, 2015 to December 31, 2019, supplemented with claims for long-term care from January 1, 2016 to December 31, 2019 for patients who reside in regions where VGZ is also accountable for their long-term care (see Box 1). Data in both datasets was combined on anonymised patient information. All research processes were determined to be in accordance with regulations regarding general data protection.

**Box 1. Brief Summary of System Characteristics of the Curative and Long-term Care in the Netherlands^22^**

**Curative Care**

Dutch inhabitants are compulsorily insured for all curative care ranging from care provided by GPs and hospital care to prescription drugs. Although taking up insurance is mandatory, inhabitants can choose their own healthcare insurance company, with four major insurer groups covering 90% of all Dutch inhabitants.

**Long-term Care**

Dutch inhabitants are automatically insured for long term care with income-related premiums. The long-term system care system covers a wide range of services delivered to patients requiring around the clock care either in their own homes or in nursing homes, including costs for permanent nursing home residency, home care, daytime activities, and medical devices. Long-term care is being arranged on a regional basis by 31 regional care offices. By law, the insurance company with the largest share of patients in a region has to arrange all care for patients in that specific region; regardless of the patient’s insurer for curative care.

----------------

Abbreviation: GPs, general practitioner.

###  Participants

 Patients with dementia were identified using specific pharmacy and care activity claims that a recent publication on dementia networks in the Netherlands found are strongly correlated with dementia^23^ (see Supplementary file 1). As dementia is a progressive condition, patients can be identified as having dementia from their first dementia care activity claim onwards. When identified, a patient’s claims data is included from the year prior to the first dementia care activity onwards. Patients below the age of 40 were excluded from the analytical cohort, in line with the aforementioned publication on dementia networks.^23^

 The cohort was subsequently divided into two groups according to whether they received care from one of the community care networks that participated in the DementiaNet programme. A GP is present in all networks, so participants were included in the intervention group if their listed GP was delivering dementia care within a community care network set up under the DementiaNet programme. The control group consisted of the remaining dementia patients – those who were not treated by a GP participating in one of the networks. The intervention setup and methods are described in more detail elsewhere.^13^

###  Outcomes

 This study focused on two primary outcome measures: hospital admission and total healthcare costs. We also included supportive (secondary) outcome measures of the two respective primary outcomes.

 For admissions, the primary outcome was the effect of the DementiaNet programme on the risk of hospital admission. The secondary outcomes related to admissions measured risks of emergency department visits, intensive care admissions, primary care crisis admissions, and admission into nursing home settings. Besides admission risks, the effect on the number of in-hospital nursing days was also studied. The methods to derive these outcomes from the claims data can be found in Supplementary file 1. Time-to-event analyses were considered relevant outcomes as well, but were excluded from our analyses due to possible inaccuracies arising from delays in submission of claims by providers^24^ and the influence of waiting times for nursing home admission.^25^

 For healthcare costs, the primary outcome studied was annual total healthcare costs across curative and long-term care per patient during the period of 2016-2019. Total annual costs were measured as the total monetary value of healthcare claims in both the curative and long-term care sector per year. Secondary outcome measures were the effects on sub-elements of total healthcare costs, being annual total care costs, primary care costs, hospital care costs, district nursing (ie, care at home outside of long-term care) costs, and pharmacy costs during the years 2015-2019 for curative care. For long-term care, annual per patient costs during the period of 2016-2019 were included.

###  Procedures and Statistical Analysis

 All data processing and analysis procedures were performed using the statistical computing program R. Data was merged on patient level for every individual treatment year between 2015-2019 on an intention-to-treat basis, meaning all health claims between 2015-2019 were included from point of diagnosis regardless of possible changes in a patient’s curative care consumption pattern as a result of, for instance, nursing home admission.

 Baseline differences between the intervention and control group were determined based on *t* tests or Wilcoxon rank sum test for continuous variables or χ^2^ tests for categorical variables. Comparisons were made for both the curative care and smaller long-term care cohort originating from this larger cohort as enrolment in long-term care data is not completely random (see Box 1). Also, temporary effects on healthcare use in the Dutch long-term care sector as a result of policy reforms in 2015^26^ could have introduced certain differences. Baseline differences between DementiaNet subgroups with different years of programme enrolment were also studied.

 Differences in outcomes between the DementiaNet group and the control group were assessed by mixed effects regression models, allowing us to control for unobserved bias arising from possible clustering of repeated measurements within patients over treatment years and practice variation between GP-practices.^27^ Only random intercepts were added since analysis of variance (ANOVA) test established that adding random slopes did not significantly improve model fits. Linearity, homogeneity of variance, and normal distribution of residuals were tested with residual plots and data was transformed accordingly if model assumptions were being violated. Being treated by a GP enrolled in a DementiaNet community care network was coded as a dichotomous variable that converted from the year of a GP’s enrolment onwards to let participants serve as their own control for healthcare use until the year of enrolment. The treatment year was included to utilise the longitudinal aspect of the data and to enable measurements of effects per treatment year within patients. We also controlled for several confounders: sex,^28,29^ age,^28,29^ cumulative multimorbidity-score,^28,30^ year of diagnosis,^28,30^ and socioeconomic status (SES). For models including long-term care data, an additional correction for region and average curative care expenses were added. The methods to derive these confounders from the claims data can be found in Supplementary file 1. Separate models were fitted for all primary and secondary outcome measures to test model assumptions and distribution.

 For models determining differences in admission risks, mixed effects logistic regression models assuming binomial distributions were used. However, as a binomial distribution did not fit the data for the number of in-hospital nursing days, a Poisson distribution was used. Similarly, the relationship between DementiaNet community care networks and nursing home admissions was assessed by logistic regression models without any mixed effects due to a lack of repeated measures since patients, once admitted, often no longer leave a nursing home and are therefore only admitted once. All models used count data of events at patient level to calculate the risk of a specific event. In case a limited number of events per patient per year resulted in models unable to accurately cluster measurements per patient, only a random intercept for GP-practice number was added and data was no longer clustered per treatment year. Age and number of co-occurring chronic diseases were rescaled into an ordinal and dichotomous variable to better fit the binary predictors and outcomes used in the logistic models.

 For healthcare costs, differences were assessed by mixed effects linear regression models assuming Gaussian distributions. Both a random intercepts per patient and GP-practice were added.

 Community care networks participating in the DementiaNet programme are currently mostly concentrated within two regions in the east and south of the Netherlands. Large differences in prices for hospital care can exist as a result of price negotiations between healthcare insurers and providers.^31^ As a sensitivity analysis for variations due to regional clustering and price negotiations, a linear mixed model containing standardised prices for hospital care activities from the Dutch healthcare authority was used. Additionally, to study the effect of DementiaNet on several types of curative care admissions at once, we performed a sensitivity analysis in which hospital admission, emergency department visit, intensive care unit admission, and primary care crisis admission were merged into a single variable “curative care admissions.”

## Results

 Curative care claims data contained 38 799 patients with dementia, and after excluding patients below the age of 40, the dataset contained 38 525 patients. Of these, 485 patients were treated by community care networks participating in the DementiaNet programme (see Figure). Long-term care data was available for 9677 patients, including 252 patients treated by community care networks (see Figure). Seventy-five individual primary care physicians amongst 31 primary care practices participating in the DementiaNet programme were identified in the claims data.

**Figure:**
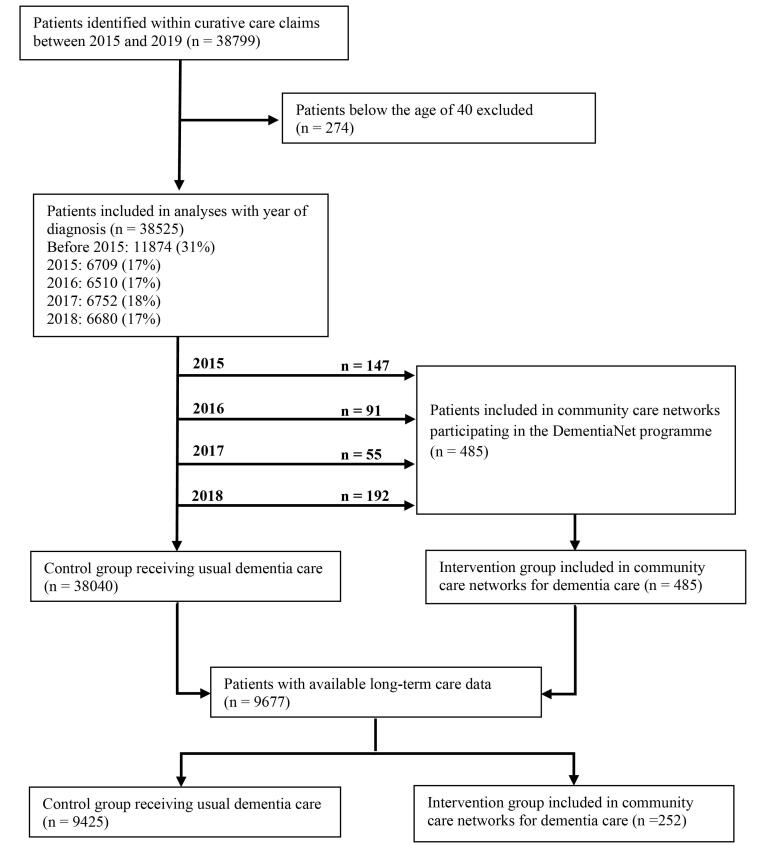
A Graphic Representation of the Composition of the Study Cohort.

Tables 1 and 2 show the descriptive statistics of the intervention group and control group. Besides age and SES, groups were comparable for most patient characteristics at baseline, including 5-year mortality rates and year of dementia diagnosis (Table 1). With respect to the long-term care claims, there was a significantly higher percentage of women in the control group and patients in the control group were significantly older. In addition, the intervention group mainly consisted of people of high and low SES category, although this was evenly distributed in the control group (see Table 2). Differences in 5-year mortality rates and year of dementia diagnosis between both groups remained non-significant. Table S2 in Supplementary file 2 shows differences in subgroups per year of DementiaNet enrolment. Groups were comparable with regards to number of comorbidities and gender, but showed differences in age, mortality during study period and SES.

**Table 1:** Comparison of Patient Characteristics Amongst Curative Care Claims Data Between Patients Included in Community Care Networks Participating in the DementiaNet Programme and the Control Group Receiving Usual Dementia Care

**Patient Characteristic**	**Intervention (n = 485)**	**Control (n = 38 040)**	***P***** Value**
Age at diagnosis (y)	76.9 (SD 9.9)	78.1 (SD 9.3)	.006^a^
Gender (%)	Male: 48.1%	Male: 46.0%	.375^b^
	Female: 52.0%	Female: 54.0%	
Comorbidities at diagnosis (n)	2.1 (SD 1.7)	2.1 (SD 1.7)	.849^a^
Mortality during study period (%)	42.7%	46.4%	.104^b^
Year of diagnosis (%)	Before 2015: 139 (29%)	Before 2015: 11 735 (31%)	.760^b^
	2015: 90 (19%)	2015: 6619 (17%)	
	2016: 85 (18%)	2016: 6425 (17%)	
	2017: 91 (19%)	2017: 6661 (18%)	
	2018: 80 (16%)	2018: 6600 (17%)	
SES (%)	Low: 36.9%	Low: 34.7%	<.001^b^
	Middle: 11.5%	Middle: 31.8%	
	High: 51.5%	High: 33.6%	

Abbreviations: SES, socioeconomic status; SD, standard deviation. ^a^ One-way ANOVA; ^b^ Pearson’s chi-squared test.

**Table 2:** Comparison of Patient Characteristics Amongst Long-term Claims Data Between Patients Included in Community Care Networks Participating in the DementiaNet Programme and the Control Group Receiving Usual Dementia Care

**Patient Characteristic**	**Intervention (n = 485)**	**Control (n = 38 040)**	***P***** Value**
Age at diagnosis (y)	79.7 (SD 8.0)	80.8 (SD 7.7)	.038^a^
Gender (%)	Male: 47.2%	Male: 37.9%	.003^b^
	Female: 52.8%	Female: 62.1%	
Comorbidities at diagnosis (n)	2.1 (SD 1.7)	2.2 (SD 1.7)	.481^a^
Mortality during study period (%)	54.0%	58.9%	.116^b^
Year of diagnosis (%)	Before 2015: 85 (34%)	Before 2015: 3667 (39%)	.440^b^
	2015: 56 (22%)	2015: 1820 (19%)	
	2016: 43 (17%)	2016: 1515 (16%)	
	2017: 42 (17%)	2017: 1378 (15%)	
	2018: 26 (10%)	2018: 1045 (11%)	
SES (%)	Low: 43.3%	Low: 29.4%	<.001^b^
	Middle: 8.2%	Middle: 38.6%	
	High: 48.5%	High: 32.0%	

Abbreviations: SES, socioeconomic status; SD, standard deviation. ^a^ One-way ANOVA; ^b^ Pearson’s chi-squared test.

Table 3 shows the relationship between inclusion in community care networks participating in the DementiaNet programme and admission risks, adjusted for comorbidity, SES, gender, and age. Patients in the DementiaNet group had lower risk of all types of admissions. Patients being treated within community care networks participating in the DementiaNet programme showed to have incurred 12% significantly less in-hospital nursing days (relative risk [RR] 0.88; 95% CI: 0.77–0.96). Hospital and intensive care admission risks showed trends for significance at the *P* <.1 level (odds ratio [OR] 0.83; 95% CI: 0.67–1.03 and OR 0.59; 95% CI: 0.34–1.01). Additional analyses per year of DementiaNet enrolment (Supplementary file 2) showed comparable results for risk of hospital admission for all years. Patients within community care networks participating in the DementiaNet programme were significantly less often admitted into nursing homes at baseline, but these differences were not significant when controlling for confounders in the linear regression model (ie, region, comorbidity, SES, gender, and age).

**Table 3:** Admission Risk Comparisons for Patients Included in Community Care Networks Participating in the DementiaNet Programme and the Control Group Receiving Usual Dementia Care, Adjusted for Comorbidity, Socioeconomic Status, Gender, and Age

**Type of Admission **	**Risk for Intervention Compared to Control (95% CI)**	***P***** Value**
Hospital admission^a^ (n = 37 205)	OR 0.83 (0.67–1.03)	.096
Emergency department visit^a^(n = 37 205)	OR 0.88 (0.72–1.08)	.234
Intensive care unit admission^b^(n = 37 205)	OR 0.59 (0.34–1.01)	.055
Number of in-hospital nursing days^c^ (n = 17 798)	RR 0.88 (0.77–0.96)	<.01
Primary care emergency admissions^c^ (n = 28 792)	OR 0.75 (0.43–1.32)	.320
Admission to nursing home setting^d^ (n = 9677)	OR 0.96 (0.80–1.15)	.656

Abbreviations: CI, confidence interval; RR, relative risk; OR, odds ratio. ^a^Mixed effects logistic regression model with binary distribution (yes/no) and correction for treatment year. ^b^Mixed effects logistic regression model with binary distribution (yes/no). ^c^Mixed effects logistic regression model with Poisson distribution (no. inpatient days). ^d^Logistic regression model with binary distribution (yes/no) and correction for treatment year.

Table 4 shows the results of the linear mixed-model comparisons of patients included in community care networks and the control group related to healthcare costs, adjusted for comorbidity, year of diagnosis, SES, gender, and age. DementiaNet community care network inclusion was associated with a non-significant decrease of 3.6% in annual total healthcare costs per patient (*P* =.303; -10.4–3.2). Additional analyses per year of DementiaNet enrolment (Supplementary file 2) showed non-significant results across all years as well. For hospital care (over 35% of total costs for curative care between 2015-2019), a significant decrease of 19.7% (95% CI: -7.6–30.3) in annual costs was found. Primary care costs (4% of total costs for curative care between 2015-2019) showed a significant increase of 10.2% (95% CI: 2.3–18.6) when compared to controls. All other cost categories showed no significant differences between patients included in community care networks participating in the DementiaNet programme and the control group.

**Table 4:** Annual Cost Comparisons for Patients Included in Community Care Networks Participating in the DementiaNet Programme and the Control Group Receiving Usual Dementia Care, Adjusted for Comorbidity, Year of Diagnosis, Socioeconomic Status, Gender, and Age

**Cost Category**	**Change Per Year for Intervention Compared to Control (95% CI)**	***P***** Value**
Total healthcare costs^a^ (n= 9378)	- 3.6% (-10.4% – +3.2%)	.303
Total curative care costs^b^ (n = 38 525)	- 3.0% (-13.0% – +8.2%)	.58
Hospital care costs^b^ (n = 37 205)	- 19.7% (-7.6 – -30.3%)	<.01
Primary care costs^b^ (n = 38 267)	+ 10.2% (+2.3% – +18.6%)	.010
District nursing care costs^b^ (n = 28 792)	+ 0.10% (-14.7% – +18.5%)	.949
Pharmaceutical costs^b^ (n= 37 751)	- 4.1% (-11.7% – +4.1%)	.318
Long-term care costs (n= 9677)	+1.0% (-6.5% – +8.6%)	.789

Abbreviation: CI, confidence interval. ^a^ Curative and long-term care combined. ^b^ Log-transformed outcome variable because of skewed distribution.

 Results for the sensitivity analyses are shown in Supplementary file 3. The outcomes of the model with standardised prices for hospital care and the model with cost prices were found to be almost identical (see Table S4 in Supplementary file 3). The effect of DementiaNet on all curative care admissions combined in one variable showed comparable results with regards to the protective trend of DementiaNet on admissions shown in Table 3 as well (OR 0.84; 95% CI: 0.70–1.02) (see Table S5 in Supplementary file 3).

## Discussion and Conclusion

 This study aimed to assess the long-term impact of DementiaNet’s community care network approach on admission risks and healthcare for patients with dementia and demonstrates its potential as a strategy to achieve more sustainable and integrated dementia care, resulting in beneficial effects for both admission risks and hospital care costs over a period of up to five years. Our results indicate that participation in the DementiaNet programme could result in fewer hospitalisations but could also prevent more serious intensive care admissions and admissions into nursing homes. This study also shows that, besides reducing admission risks and in-hospital nursing days, the DementiaNet programme does not seem to increase total healthcare costs and can reduce annual hospital care costs by as much as 19.7%, which, in absolute terms, far exceeds the 10.2% increase in primary care costs.

 A recent meta-analysis of the effects of psychosocial interventions concludes that care coordination strategies could decrease nursing home admission rates of patients with dementia.^32^ Although this seems in line with our results, both studies on which this pooled effect is based concern unidimensional, hospital-initiated case management-like programmes instead of community network approaches.^32^ All other strategies in this meta-analysis, both unidimensional and multidimensional, failed to show results.^32^ The fact that our study showed favourable results (where others do not) can be attributed to two factors. Firstly, many of the unidimensional interventions in the meta-analysis focus solely on improving one aspect of care for people with dementia (eg, formation of a network).^32^ The DementiaNet programme, on the other hand, is based on the theory that integrated care can only be achieved by focusing simultaneously on multiple aspects of integrated dementia care.^13^ This approach is more likely to create networks in which sustainable interprofessional collaboration is maintained over a longer period of time.^13^ Secondly, for those programmes that apply a multidimensional strategy, results come mostly from studies in the controlled setting of a randomised control trial (RCT) or in small uncontrolled groups, both only followed for a short period of time. However, dementia care in daily practice settings is likely to be different from that in RCT settings^33^ and the formation of sustainable collaborations in networks requires time before beneficial effects are achieved.

 Thanh et al show similar results for care networks in Alberta, Canada in their small-scale observational study: hospital care costs decreased by increasing the use of community services.^19^ The observed increase in primary care costs found in our study may be attributed to the same mechanisms. Stricter monitoring of patients and increased sharing of information on a patient’s health status between providers could result in earlier detection of increased frailty and health risks. Although this might result in some additional visits to primary care services, it prevents more costly inpatient hospital admissions. Another study found increased healthcare utilisation for certain types of outpatient hospital services in Germany.^20^ Although this seems to contradict our results, Germany is known to have relatively high numbers of consultation and hospitalisations due to the absence of a primary care-based gatekeeping system,^34^ while GPs in the Netherlands serve as strong gatekeepers.^22^ The additional outpatient visits found in Germany are, therefore, likely to be caused by the same mechanisms that cause an increase in primary care visits in the Dutch and Canadian setting. Such differences in outcomes depending on health system characteristics suggest that networks should be tailored towards the systems in which they are being implemented.

 Our study has several strengths, due to its use of claims data instead of RCT data or other types of observational data. This gives an unbiased, long-term, and comprehensive overview of the healthcare use of a certain group of patients. By looking at both curative and long-term care data, our study provides insight into almost the entire care trajectory of patients with dementia, ensuring that savings in one sector are not achieved by shifting costs to another sector. Mandatory and automatic enrolment in the curative and long-term care sector in the Netherlands ensures that all eligible patients of DementiaNet were included. As our cohort is representative of the Dutch population,^35^ these results are a reliable indication of what the DementiaNet programme could achieve throughout the Netherlands.

 Our study also has some limitations. Claims data contain no direct information on actual health status and diagnosis on the patient level, meaning we were unable to identify diagnosed patients or directly correct for unobserved health status. We tried to minimise errors of deriving this information from healthcare use in several ways by following a validated method^23^ to identify patients with dementia based on specific pharmacy and care activity claims data (Supplementary file 1). In correcting for differences in disease severity and health status, we included co-variates that are known to be valid proxies for disease severity and progression of patients with dementia, such as age, number of co-morbidities, mortality, and year of diagnosis. Differences between groups for these proxies were mostly found to be non-significant (Tables 1 and 2).

 Our entire cohort encompasses a large number of over 38 525 individuals, yet only a part of them participated in the DementiaNet programme’s community care networks. Such an unbalanced dataset could bias the results. Also, as participation was voluntary, there might have been unobserved baseline differences between participating primary care practices and the national control group. Although this would justify a before-after comparison, earlier studies show that such analyses can result in significant overestimations of outcomes.^36^ Alternatively, a mixed model design was selected to correct for this by allowing clustering of data within practices and control for possible unobserved practice variation at this level.

 Finally, DementiaNet networks have not been implemented throughout the Netherlands, but are clustered in a few regions and implemented in a stepwise approach across several years. This could lead to baseline differences between groups because of unmeasurable demographic differences between regions and short or long-term effects. Even though we did find some differences at baseline for the entire cohort and between different years of enrolment, groups were comparable for most proxies of health status at baseline. Moreover, the clustering per patient and correction for regional health office and SES in our mixed models should reduce the impact of such potential differences. The fact that results for total healthcare costs and hospital admissions across years of DementiaNet enrolment are comparable also does not indicate any differences in short or long-term effects.

 This study demonstrates that DementiaNet’s approach, with formation of local community care networks, is most likely effective in lowering admission risks and hospital care costs for patients with dementia. Combined with earlier favourable results on quality of care and network collaboration shown in several studies,^14-16^ the DementiaNet programme seems to be a value-adding initiative that is able to generate higher quality of care at the same costs. Although DementiaNet’s approach specifically focussed on the setup of community care networks for patients with dementia, this programme and its networks may be able to add value for a larger group of patients. After all, professionals active in DementiaNet networks share various other patient groups with complex care needs that could benefit from care coordination through community care networks, like frail older adults or patients in need of palliative care.

 Within the Netherlands, the results of this study can be a direct encouragement to set up similar networks throughout the country. Until now, setting up a network within the DementiaNet programme was a bottom-up approach that was initiated by network participants on a voluntary basis. The results of this study justify an approach in which broader implementation of such networks is encouraged. Guaranteed, long-term financial support and inclusion in nationwide guidelines on dementia care can assist this process of national uptake. Networks could be funded by redistributing funds from the identified 20% savings in hospital care towards primary care, but such processes are highly complex and require agreements between payers and providers across all levels of care. Internationally, policy-makers may want to actively encourage the establishment of similar DementiaNet community care networks through comparable mechanisms. However, international differences in healthcare systems requires networks to be tailored to the local needs in dementia care and evaluated separately.

## Acknowledgements

 We thank Esther Hendriks and Marit Tanke (VGZ) for their perseverance in realising approval and execution of the merging of curative and long-term care data. Also, we thank all those at VGZ and at the Department of Geriatrics who have helped us in the design and execution of the research, especially Marieke Perry and Minke Nieuwboer. Lastly, thanks to Nicholas Crawford for his phenomenal help in proofreading and language checking of this manuscript.

## Ethical issues

 A recognized medical ethics review committee determined that this research is in accordance with the Declaration of Helsinki and exempted from the Dutch law on medical scientific research in humans (application number: 2020-6201).

## Competing interests

 All authors have completed the disclosure form and declare: financial support from the Dutch Ministry of Health, Welfare and Sport for the submitted work for TER, FMK, SAvD, and PPJ. Financial support was given to the research group – not directly to researchers. MFM reported receiving personal fees from Coöperatie Volksgezondheidszorg (VGZ).

## Funding

 This work was supported by the Dutch Ministry of Health, Welfare and Sport (VWS). Whilst VWS provided funds for the research, it was not actively involved in any part of the design and conduct of the study.

## Supplementary files


Supplementary file 1. Selection Criteria & Methods for Deriving Admission Risk Outcomes and Confounders From Claims Data.

Supplementary file 2. Comparison of Baseline Characteristics and Outcomes Per Year of DementiaNet Entry.

Supplementary file 3. Sensitivity Analyses.
