# Supplementary material for: Effects of DementiaNet’s Community Care Network Approach on Admission Rates and Healthcare Costs: A Longitudinal Cohort Analysis
Source: Int J Health Policy Manag. 2023 Oct 28;12:7700. doi: 10.34172/ijhpm.2023.7700 (PMC10699814; doi:10.34172/ijhpm.2023.7700)
Supplement: Supplementary file 1 — Selection Criteria & Methods for Deriving Admission Risk Outcomes and Confounders From Claims Data. [file ijhpm-12-7700-s001.pdf]

Article title: Effects of DementiaNet's Community Care Network Approach on Admission Rates and Healthcare Costs: A Longitudinal Cohort Analysis

Journal name: International Journal of Health Policy and Management (IJHPM)

Authors' information: Toine EP Remers[1]*, Florien M. Kruse[1], Simone A. van Dulmen[1], Dorien L. Oostra[2], Martijn FM Maessen[3], Patrick PT Jeurissen[1], Marcel GM Olde Rikkert[2,4]

[1]Radboud university medical center, Scientific center for quality of healthcare (IQ healthcare), Nijmegen, The Netherlands.

[2]Radboud university medical center, Department of Geriatric Medicine, Nijmegen, The Netherlands.

[3]Coöperatie Volksgezondheidszorg, Business intelligence services, Arnhem, The Netherlands.

[4]Radboud university medical center, Donders Institute for Brain, Cognition and Behaviour, Department of Geriatric Medicine, Radboud Alzheimer Centre, Nijmegen, The Netherlands.

***Correspondence to:** Toine EP Remers; Email: toine.remers@radboudumc.nl

Citation: Remers TE, Kruse FM, van Dulmen SA, et al. Effects of DementiaNet's community care network approach on admission rates and healthcare costs: a longitudinal cohort analysis. Int J Health Policy Manag. 2023;12:7700. doi:10.34172/ijhpm.2023.7700

Supplementary file 1. Selection Criteria & Methods for Deriving Admission Risk Outcomes and Confounders From Claims Data

A: Selection criteria for participants diagnosed with dementia

Table S1: The indicator set developed by Vektis to distinguish patients with and without d*ementia in the claims dataset*

**Sector**	**Criterium for selection**
Medication (2014-2019)	- Galantamine (N06DA04)<br>- Memantine (N06DX01)<br>- Rivastigmine (N06DA03)<br>- Donepezil (N06DA02, N06DA52, N06DA53)
Hospital care (2014-2019)	- 0401 (Dementia syndromes) at Neurology (0330)<br>- 0091 (Memory problems and dementia) at Internal Medicine (code: 0313)<br>- 0242 (Memory problems and dementia) at Clinical geriatrics (0335)
Mental health care (2014-2019)	-007, Delirium, dementia, amnestic and dementia causing disorders

B: Methods for deriving admission risk outcomes

A patient was marked as having a hospital admission for every health claim that was accompanied by at least one nursing day. An emergency department visit was identified based on specific referral codes of health claims that indicate a patient has been referred to the emergency department or has decided to be refer him/herself. Intensive care admission was determined based on specific claim codes associated with patients admitted to intensive care units (190130 through 190133, 190150 through 190156, 190158, and 190219). The effects on the number of in-hospital nursing days were determined by counting the total number of days per year on which a patient was hospitalised. To determine this, the number of nursing days for each hospitalisation was derived from the health claim's description and then these were added together up to a maximum of 365 days per year. Admission into a nursing home was determined based on the type of long-term care a patient received. From the moment a patient received packages that could only be delivered within nursing home settings, a patient was identified as being admitted.

C: Methods for deriving confounders

Sex, age, and year of diagnosis were directly extracted from the data. Socioeconomic status was determined based on neighbourhood-dependent estimates from the Netherlands Institute for Social Research (SCP) linked to patient's postal code information (first four digits). Scores were subsequently divided into the classification 'low', 'moderate', or 'high'. Co-occurring chronic diseases of patients were identified based

on pharmacy claims[1]. A composite measure for multimorbidity was formed by summing the number of identified co-existing chronic conditions. For models including long-term care data, two additional covariates were added. Long-term care in the Netherlands is organised on a regional basis by regional offices, resulting in differences in policy and capacity between these regions. To correct for such potential differences, the four-digit postal code of VGZ's seven regional offices were included in our dataset. Subsequently, patients were clustered based on having the same regional office number. Also, it is likely that a patient with high costs in curative care will also incur high long-term care costs, average costs of a patient within curative care during the period 2015-2019 was also controlled for during analysis of long-term care data.

References

1. Huber CA, Szucs TD, Rapold R, et al. Identifying patients with chronic conditions using pharmacy data in Switzerland: an updated mapping approach to the classification of medications. *BMC public health* 2013;13(1):1-10.
