# Supplementary material for: Effects of DementiaNet’s Community Care Network Approach on Admission Rates and Healthcare Costs: A Longitudinal Cohort Analysis
Source: Int J Health Policy Manag. 2023 Oct 28;12:7700. doi: 10.34172/ijhpm.2023.7700 (PMC10699814; doi:10.34172/ijhpm.2023.7700)
Supplement: Supplementary file 2 — Comparison of Baseline Characteristics and Outcomes Per Year of DementiaNet Entry. [file ijhpm-12-7700-s002.pdf]

Article title: Effects of DementiaNet's Community Care Network Approach on Admission Rates and Healthcare Costs: A Longitudinal Cohort Analysis

Journal name: International Journal of Health Policy and Management (IJHPM)

Authors' information: Toine EP Remers[1]*, Florien M. Kruse[1], Simone A. van Dulmen[1], Dorien L. Oostra[2], Martijn FM Maessen[3], Patrick PT Jeurissen[1], Marcel GM Olde Rikkert[2,4]

[1]Radboud university medical center, Scientific center for quality of healthcare (IQ healthcare), Nijmegen, The Netherlands.

[2]Radboud university medical center, Department of Geriatric Medicine, Nijmegen, The Netherlands.

[3]Coöperatie Volksgezondheidszorg, Business intelligence services, Arnhem, The Netherlands.

[4]Radboud university medical center, Donders Institute for Brain, Cognition and Behaviour, Department of Geriatric Medicine, Radboud Alzheimer Centre, Nijmegen, The Netherlands.

*__Correspondence to:__ Toine EP Remers; Email: toine.remers@radboudumc.nl

Citation: Remers TE, Kruse FM, van Dulmen SA, et al. Effects of DementiaNet's community care network approach on admission rates and healthcare costs: a longitudinal cohort analysis. Int J Health Policy Manag. 2023;12:7700. doi:10.34172/ijhpm.2023.7700

Supplementary file 2. Comparison of Baseline Characteristics and Outcomes Per Year of DementiaNet Entry

Table 1: Comparison of characteristics between patients with different years of enrollment in community care networks participating in the DementiaNet programme

Patient characteristic	2015 (147)	2016 (91)	2017 (55)	2018 (192)	*P* value
**Age at diagnosis** (years)	76.2 (SD 10.39)	79.2 (SD 8.91)	74.9 (SD 9.48)	77.0 (SD 9.87)	**.040**±
**Gender** (%)	Male: 46%<br>Female: 54%	Male: 54%<br>Female: 46%	Male: 40%<br>Female: 60%	Male: 49%<br>Female: 51%	.41*
**Comorbidities at diagnosis** (n)	2.1 (SD 1.7)	2.3 (SD 1.8)	2.2 (SD 1.4)	1.9 (SD 1.6)	.21±
**Mortality during study period** (%)	41%	60%	36%	38%	**.002***
**SES** (%)	Low: 20%<br>Middle: 8.8%<br>High: 71%	Low: 67%<br>Middle: 7.7%<br>High: 25%	Low: 25%<br>Middle: 29%<br>High: 45%	Low: 39%<br>Middle: 10%<br>High: 51%	**<.001***
Abbreviation: SD, standard deviation.<br>±: Kruskal-Wallis rank sum test<br>*: Pearson's Chi-squared test					

Table 2: Comparison of primary outcome measures total healthcare costs and hospital admissions between patients with different years of enrollment in community care networks participating in the DementiaNet programme

Comparison of primary outcome measures per year of DementiaNet entry

Outcome	2015 (147) (95% CI)	2016 (91) (95% CI)	2017 (55) (95% CI)	2018 (192) (95% CI)
**Change in annual total healthcare costs (%)**	-3.1% (-21.0% – +14.9%)	+1.7% (-11.1% – +14.4%)	+9.1% (-3.4% – +21.6%)	-7.3% (-16.7% – +2.2%)
**Risk for hospital admissions (OR)**	0.74 (0.36-1.52)	0.96 (0.59-1.55)	0.75 (0.49-1.15)	0.79 (0.57-1.10)
Abbreviation: CI, confidence interval.				
