# Supplementary material for: Effects of DementiaNet’s Community Care Network Approach on Admission Rates and Healthcare Costs: A Longitudinal Cohort Analysis
Source: Int J Health Policy Manag. 2023 Oct 28;12:7700. doi: 10.34172/ijhpm.2023.7700 (PMC10699814; doi:10.34172/ijhpm.2023.7700)
Supplement: Supplementary file 3 — Sensitivity Analyses. [file ijhpm-12-7700-s003.pdf]

Article title: Effects of DementiaNet's Community Care Network Approach on Admission Rates and Healthcare Costs: A Longitudinal Cohort Analysis

Journal name: International Journal of Health Policy and Management (IJHPM)

Authors' information: Toine EP Remers[1]*, Florien M. Kruse[1], Simone A. van Dulmen[1], Dorien L. Oostra[2], Martijn FM Maessen[3], Patrick PT Jeurissen[1], Marcel GM Olde Rikkert[2,4]

[1]Radboud university medical center, Scientific center for quality of healthcare (IQ healthcare), Nijmegen, The Netherlands.

[2]Radboud university medical center, Department of Geriatric Medicine, Nijmegen, The Netherlands.

[3]Coöperatie Volksgezondheidszorg, Business intelligence services, Arnhem, The Netherlands.

[4]Radboud university medical center, Donders Institute for Brain, Cognition and Behaviour, Department of Geriatric Medicine, Radboud Alzheimer Centre, Nijmegen, The Netherlands.

***Correspondence to:** Toine EP Remers; Email: toine.remers@radboudumc.nl

Citation: Remers TE, Kruse FM, van Dulmen SA, et al. Effects of DementiaNet's community care network approach on admission rates and healthcare costs: a longitudinal cohort analysis. Int J Health Policy Manag. 2023;12:7700. doi:10.34172/ijhpm.2023.7700

Supplementary file 3. Sensitivity Analyses

Table S4: Differences in hospital care cost comparisons for patients included in community care networks participating in the DementiaNet programme and the control group receiving usual dementia care - standardised vs cost prices

**Outcome**	**Change per year in hospital care costs (95% CI)**	**p-value**
**Hospital care – contracted prices[1]** (n = 37,205)	- 19.71 % (-7.58 – -30.26%)	< 0.01
**Hospital care – standardised prices[1]** (n = 37,205)	- 19.74 % (-7.68 – -30.23%)	< 0.01
[1]: Log-transformed outcome variable because of skewed distribution		

Table S5: Admission risk comparison for all curative care admissions combined for patients included in community care networks participating in the DementiaNet programme and the control group receiving usual dementia care, adjusted for comorbidity, socioeconomic status, gender, and age.

**Type of admission**	**Risk for intervention compared to control (95% CI)**	***P* value**
**Curative care admission***(n = 37,816)	OR 0.84 (0.70 – 1.02)	0.078
*: Mixed effects logistic regression model with binary distribution (yes/no) and correction for treatment year		
